# Clinic—Buffalo Hospital Sisters of Charity

**Published:** 1909-05

**Authors:** L. G. Hanley


### CLINICAL REPORTS.

### Clinic—Buffalo Hospital Sisters of Charity

L. G. HANLEY, M. D,

REPORTED BY

JOHN STOWE, M. D., Interne.

### EXTRAUTERINE PREGNANCY—OBSTRUCTION OF BOWEL—A RUPTURE OF SAC.

Case I.—February 2, 1909, Mrs. B., age 34; number of children, 4; no miscarriages; youngest child one year old, has had no sickness. Has always been regular with her menstrual periods till November 1, 1908, when she had menorrhagia, which lasted about three weeks. Menstruated December 20, 1908. Says she has been failing since October. Was admitted to hospital February 2, 1909, suffering from obstruction of the bowel. There had been no movement for six days, abdomen distented, tender, tympanitic, vomiting, and looks very anemic. Enemata were given high—Nobles—and syrupus fuscus and water, no effect. Examination per vaginam: a soft boggy mass, circumscribed and filling the pelvic cavity. Hemoglobin, 30; white corpuscles, 10.600; patient prepared for operation. An opening was made in the posterior culdesac, when a dark sanguineous fluid escaped: on further examination the bowels and omentum were found adhered together by clots. A suprapubic incision was made and pelvis cleaned of all clots and membrane. The right tube was ruptured about the middle third, and from appearances must have contained a fetus of two months gestation, which, however, was not found, but might have been lost among the clots. There was over two quarts of blood in the abdominal cavity. It was necessary to separate and wipe several feet of bowel, so matted were they from adhesions. The uterus was twice its normal size; the right tube was removed, but the ovary left in place as it appeared healthy. Abdominal cavity flushed with normal salt solution. A vaginal drain was inserted, "patient placed in Fowler's position and stimulants administered. Patient improved and left the hospital March 18, 1909, cured.

### INTERSTITIAL ECTOPIC GESTATION.

Case II.—Miss W., admitted to hospital March 1, 1909, discharged March 20, 1909, age 22; family history, good; personal history: began to menstruate at twelve years of age, regular every twenty-eight days. Present attack began January 28, 1909, with sharp pain in the right side; this was accompanied with nausea and vomiting, and she was compelled to remain home in bed for four weeks. Pain that was at first confined to the right side has become general over the abdomen.

Examination shows a tumor on the right side connected with the tube and uterus; patient prepared for operation, and on opening the abdominal cavity a tumor 4 inches long and 2 inches thick, with 4 inches of ileum attached was brought into view. Bowel relieved and tumor removed, which showed that the sac with the product of conception was partially in the uterus. Cavity in the uterus was closed, ovary not removed. Patient made a quick recovery.

ECTOPIC GESTATION.

CASE III.—Mrs. M., age 20, family history good. Personal history: no diseases except the ordinary diseases of childbirth. Menstrual period began at fifteen years of age, regular every month till December 5, 1908; was seen February 7, 1909, at 6 P.M., by Dr. P. H. Hourigan, who found her suffering from shock, pain in right side and all symptoms of internal hemorrhage. When admitted to hospital one hour later was pulseless, with rapid and shallow breathing; hemoglobin, 40, and in extremis. Patient given an anesthetic, abdomen opened quickly, when right tube was found ruptured. This was immediately clamped and hypodermoclysis, normal salt solution given. The abdomen was filled with blood, tube removed, but ovary left in place. Patient left the hospital in three weeks.

